# A88 ADVERSE EVENTS ASSOCIATED WITH ENDOSCOPIC RETROGRADE CHOLANGIOPANCREATOGRAPHY: A SYSTEMATIC REVIEW AND META-ANALYSIS

**DOI:** 10.1093/jcag/gwad061.088

**Published:** 2024-02-14

**Authors:** H Guo, K Bishay, Z Meng, Y Ruan, D Brenner, N Forbes

**Affiliations:** Medicine, University of Calgary, Calgary, AB, Canada; Royal Alexandra Hospital, Edmonton, AB, Canada; Medicine, University of Calgary, Calgary, AB, Canada; Medicine, University of Calgary, Calgary, AB, Canada; Medicine, University of Calgary, Calgary, AB, Canada; Medicine, University of Calgary, Calgary, AB, Canada

## Abstract

**Background:**

Endoscopic retrograde cholangiopancreatography (ERCP) is a widely utilized procedure for diagnosing and managing various biliary and pancreatic disorders. Despite its established effectiveness, ERCP is associated with a total adverse event (AE) rate exceeding 10%. However, existing literature lacks a comprehensive synthesis of incidence rates pertaining to specific or overall AEs following ERCP procedures.

**Aims:**

We performed separate systematic reviews and meta-analyses of (1) data from randomized controlled trials (RCTs) and (2) data from observational studies to evaluate the incidence of AEs following ERCPs in adult patients.

**Methods:**

Two separate systematic literature searches were conducted to identify ERCP AE rates in RCTs and observational studies published between 2000 and 2021, inclusive. Abstracts underwent independent assessment to identify studies for full-text review and subsequent data extraction. DerSimonian and Laird random effects meta-analyses were applied to determine pooled incidence rates of individual post-ERCP AEs, accompanied by 95% confidence intervals (CIs). The Newcastle-Ottawa Scale (NOS) and Risk of Bias 2 (ROB2) tool were used for quality assessment of observational studies and RCTs respectively.

**Results:**

Our analysis incorporated 242 RCTs and 143 observational studies. Among RCTs, the pooled incidences of post-ERCP pancreatitis (PEP) in patients with native and non-native papillae were 6.9% (CI 6.2% to 7.6%) and 6.1% (CI 5.3% to 7.1%), respectively. In observational studies, the pooled PEP incidences were 5.0% (CI 4.0% to 6.1%) for patients with native papillae and 4.2% (CI 3.6% to 4.8%) for patients with non-native papillae. The incidences of bleeding for patients with native papillae were 1.6% (CI 1.3% to 2.0%) and 2.2% (CI 1.2% to 3.9%) in RCTs and observational studies, respectively. The incidences of perforation for patients with native papillae were 0.3% (CI 0.2% to 0.4%) in RCTs and 0.5% (CI 0.3% to 0.7%) in observational studies. The incidence of cholangitis was 1.4% (CI 1.1% to 1.9%) in RCTs and 1.1% (CI 0.7% to 1.7%) in observational studies.

**Conclusions:**

This meta-analysis offers comprehensive insights into the incidence of ERCP-associated AEs from 2000 to 2021, both in idealized study settings where procedures are performed by experts, and in ‘real world’ settings. More precise estimates of ERCP-related AEs can help facilitate patient consent, manage appropriate patient expectations, and enhance peri-procedural care.

**Funding Agencies:**

None

